# Myocardial injury in systemic lupus erythematosus and lupus nephritis: a silent threat

**DOI:** 10.1093/ckj/sfag305

**Published:** 2026-09-02

**Authors:** Eleni Moysidou, Maria-Anna Bazmpani, Alexandros Kallifatidis, Dimitrios Kotzadamis, Efstathios Pagourelias, Theodoros Karamitsos, Maria Stangou, Eleni Frangou

**Affiliations:** School of Medicine, Aristotle University of Thessaloniki, Thessaloniki, Greece; Nefrozoi Medical Center, Nicosia, Cyprus; Department of Cardiology, AHEPA Hospital, Aristotle University of Thessaloniki, Thessaloniki, Greece; St Luke’s Hospital, Panorama, Thessaloniki, Greece; Third Department of Cardiology, Hippokration Hospital, Aristotle University of Thessaloniki, Thessaloniki, Greece; School of Medicine, Aristotle University of Thessaloniki, Thessaloniki, Greece; Third Department of Cardiology, Hippokration Hospital, Aristotle University of Thessaloniki, Thessaloniki, Greece; School of Medicine, Aristotle University of Thessaloniki, Thessaloniki, Greece; Department of Cardiology, AHEPA Hospital, Aristotle University of Thessaloniki, Thessaloniki, Greece; School of Medicine, Aristotle University of Thessaloniki, Thessaloniki, Greece; First Department of Nephrology AUTH, Hippokration Hospital, Thessaloniki, Greece; Department of Nephrology, Limassol General Hospital, State Health Services Organization, Limassol, Cyprus; University of Nicosia Medical School, Nicosia, Cyprus; Department of Nephrology, Aretaieion Hospital, National and Kapodistrian University of Athens Medical School, Athens, Greece

**Keywords:** cardiac magnetic resonance, cardiovascular disease, lupus nephritis, myocardial involvement, systemic lupus erythematosus

## Abstract

Systemic lupus erythematosus (SLE) is associated with significant cardiovascular morbidity, with myocardial involvement representing one of the most clinically significant yet underrecognized cardiac manifestations, largely due to its frequently subclinical presentation and diagnostic complexity. Lupus nephritis (LN), a common and clinically significant manifestation of SLE, is further associated with increased cardiovascular risk, reflecting a dynamic cardiorenal interaction driven by persistent immune activation. Recent advances in cardiovascular imaging techniques have facilitated the early detection of myocardial injury in SLE, improving diagnostic accuracy and enabling timely intervention, but have also uncovered the increased possibility of subclinical, underdiagnosed cases. Herein, this review focuses on myocardial disease in SLE, outlining the diagnostic tools available for early detection of myocardial complications.

## INTRODUCTION

Systemic lupus erythematosus (SLE) is a prototypical chronic autoimmune disease whose fundamental pathogenic mechanism is considered to be the disrupted communication between innate and adaptive immunity, resulting in the activation of immune mechanisms (cytokine and antibody production) and ultimately damage to multiple organs [1]. Among affected organs, cardiac manifestations in SLE can occur at any stage of disease progression and may affect all cardiac structures [2], serving as a major determinant of morbidity and mortality, mainly in patients with kidney involvement and young individuals [3].

The pathogenesis of cardiac involvement in SLE is complex and multifactorial (Fig. 1). Dysregulation of type I interferon signalling, innate immune activation, and adaptive immune responses leads to endothelial dysfunction, immunothrombosis, accelerated atherosclerosis, and myocardial inflammation [4–8].

**Figure 1:**
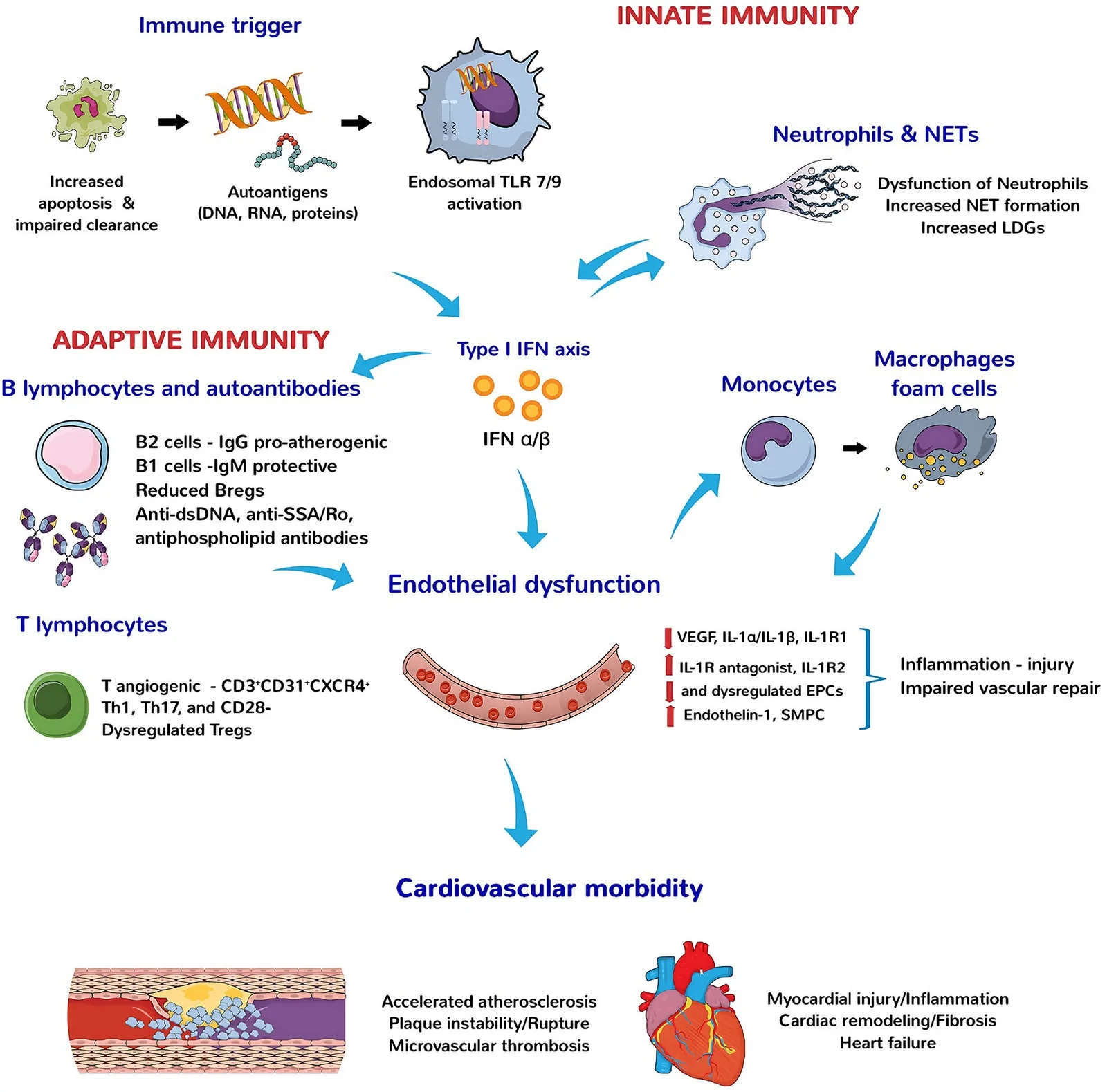
Immune-mediated mechanisms linking SLE to cardiovascular disease. Impaired clearance of apoptotic material triggers activation of endosomal Toll-like receptors (TLRs) and a type I interferon (IFN)-driven inflammatory response. Innate immune pathways—including NET formation, low-density granulocytes, and macrophage activation—promote endothelial dysfunction, oxidative stress, and vascular inflammation. Type I IFNs further impair endothelial repair and angiogenesis, contributing to a pro-atherogenic environment. Adaptive immune responses, through autoantibody production and pro-inflammatory T-cell activity, amplify vascular injury and immunothrombosis. These interconnected mechanisms ultimately lead to accelerated atherosclerosis, thrombosis, and myocardial inflammation, underlying the increased cardiovascular burden in patients with SLE. Created in BioRender. MOYSIDOU, E. (2026) https://BioRender.com/fcmmtww Abbreviations: Anti-dsDNA, anti double-stranded DNA antibodies; anti-SSA/Ro, anti-Sjögren’s syndrome A antibodies; Bregs, B regulatory cells; EPCs, endothelial progenitor cells; IL, interleukin; IL-R, interleukin receptor; SMPC, smooth muscle progenitor cells; Th1, T-helper 1; Th17, T-helper 17; Tregs, T regulatory cells; VEGF, vascular endothelial growth factor.

However, although overlapping immune-mediated pathways contribute to cardiac involvement in SLE, the underlying mechanisms are not uniform and likely differ across specific manifestations. Libman–Sacks endocarditis (LSE) represents a valvular process driven predominantly by endothelial injury and immune complex deposition—often in the context of antiphospholipid antibodies—resulting in sterile verrucous vegetations [9], whereas myocarditis is primarily characterized by inflammatory and immune-mediated injury, including perivascular immune complex deposition, local complement activation, and recruitment of mononuclear cells, leading gradually to fibrosis [10]. Given this mechanistic heterogeneity, early and sensitive detection of cardiac involvement is essential. Several cardiac abnormalities, particularly those involving the myocardium, may remain subclinical and are often undetectable by clinical examination or conventional diagnostic tools. In recent years, cardiac magnetic resonance (CMR) has emerged as a valuable tool for identifying early, subclinical lesions that would otherwise go unnoticed and progress over time [11]. Thus, timely recognition and management of cardiac involvement are essential to improving outcomes and reducing cardiovascular mortality in SLE.

This review focuses on cardiac involvement in SLE, with particular emphasis on myocardial disease, the challenges and the evolving role of CMR in early diagnosis and risk stratification, and the contribution of lupus nephritis (LN) to cardiovascular risk.

## SEARCH STRATEGY AND STUDY SELECTION

This review was conducted as a narrative review and is based on a structured literature search performed in PubMed. No predefined time restriction was applied, and studies published up to August 2025 were considered. Only articles published in the English language were included. The search strategy incorporated combinations of the following keywords: ‘systemic lupus erythematosus’, ‘lupus nephritis’, ‘myocardial involvement’, ‘myocarditis’, ‘cardiomyopathy’, ‘cardiac magnetic resonance’, ‘CMR’, ‘subclinical myocardial disease’, ‘myocardial fibrosis’, and ‘cardiovascular involvement’. Original studies involving human subjects, including cohort studies, case–control studies, and relevant case series, were prioritized. Review articles were also screened to identify additional pertinent publications and references. Studies were selected according to their relevance to cardiac and myocardial involvement in SLE, with particular emphasis on clinical manifestations, diagnostic approaches, and imaging findings. Studies evaluating CMR and cardiovascular involvement in patients with LN were included whenever available. However, evidence specifically addressing LN as a distinct subgroup remains limited, with most published data derived from broader SLE populations. This review was designed to provide a comprehensive narrative synthesis of the available evidence rather than a systematic review or meta-analysis.

## CARDIAC INVOLVEMENT IN SLE

Multiple components of the heart, including the pericardium, endocardium, cardiac valves, myocardium, the conduction system, and coronary arteries, can be affected in SLE [12, 13]. The American College of Rheumatology (ACR) classification criteria for SLE have included pericardial involvement—most commonly presenting as pericarditis—as the only cardiac manifestation in SLE presenting in the form of serositis. However, this represents just one aspect of the broader spectrum of cardiovascular pathology seen in the disease [14]. Other cardiac manifestations are not uncommon and may present at any stage of the disease, either early during initial diagnosis or later with disease progression. Despite their clinical significance, cardiac manifestations are often under-recognized, highlighting the need for increased awareness and comprehensive cardiovascular assessment in patients with SLE.

Cardiac manifestations in SLE are summarized in Table 1, which describes cardiac structures involved, main pathological and clinical presentation as well as diagnostic findings.

**Table 1:** Cardiac involvement in SLE.

Cardiac structure	Pathology	Typical clinical presentation	Key physical/diagnostic findings	Major complications
**Pericardium**	Acute or chronic pericarditis	Pleuritic chest pain relieved by sitting forward; fever; dyspnoea	Pericardial friction rub; ECG with diffuse ST elevation; echocardiography showing effusion	Cardiac tamponade (hypotension, tachycardia, jugular venous distention)
**Myocardium**	Lupus myocarditis (rare, <10%)	Fatigue, exertional dyspnoea, palpitations; subtle or acute	Reduced ejection fraction; elevated cardiac enzymes; CMR with myocardial oedema	Acute heart failure, cardiogenic shock, conduction blocks, malignant ventricular arrhythmias
**Endocardium (valves)**	LSE (sterile vegetations)	Often asymptomatic; may present with dyspnoea or fatigue	Systolic murmur from mitral or aortic regurgitation; echocardiographic valvular vegetations	Valve failure, systemic embolization, ischaemic stroke
**Coronary arteries**	Accelerated atherosclerosis; coronary vasculitis; thrombosis	Typical or atypical angina; exertional dyspnoea; may be silent	Ischaemic ECG changes; elevated troponins; coronary angiography or CT angiography	Myocardial infarction, sudden cardiac death, chronic ischaemic cardiomyopathy
**Electrical conduction system**	Inflammatory or ischaemic involvement	Palpitations, syncope	ECG abnormalities (atrioventricular block, arrhythmias)	Sudden cardiac death (rare but severe cases)

Abbreviations: CMR, cardiac magnetic resonance; CT, computed tomography; ECG, electrocardiography; LSE, Libman–Sacks endocarditis; SLE, systemic lupus erythematosus.

### Pericardium

Pericarditis is the most frequent and earliest clinical indicator of cardiac complication associated with SLE. The occurrence of pericardial effusion ranges from 21% in unselected SLE cohorts to 54% in prospective echocardiographic studies, reflecting differences in ascertainment methods—systematic imaging detecting higher rates compared with clinically overt cases—as well as the predominantly hospital-based, tertiary-referral populations from which these data derive; in contrast, prevalence in healthy controls is reported at 0%–10% [12].

Pericarditis in SLE reflects the intricate interaction between innate and adaptive immunity: immune-complex deposition on the pericardium and particularly C3 deposition is crucial in inducing local inflammation, and inflammatory cell infiltration [15–17]. A ‘type I interferon signature’ tends to predominate in autoimmune pericarditis associated with SLE [18, 19]. Additional genetic predisposition further modulates the risk. Variants in TRAF3IP2, encoding the Act1 adaptor protein involved in interleukin-17 (IL-17) signalling, represent the most consistent genetic association, with the rs33980500 allele conferring an increased risk [20]. A multi-locus model incorporating MIR1279, STAT4, PTPN2, and TRAF3IP2 variants further amplifies susceptibility [21]. Complement gene dosage provides an additional mechanism, as low C4A copy number has been associated with pericarditis in juvenile SLE (OR 5.54, *P* = .016), likely reflecting impaired immune-complex clearance [22]. Other variants, including P2RX7 and MBL2, have also been implicated, although these findings require further validation [23].

The clinical spectrum ranges from subclinical, asymptomatic effusions to symptomatic acute or recurrent pericarditis. Chronic inflammation may lead to fibrotic thickening of the pericardium and constrictive pericarditis (Fig. 2).

**Figure 2:**
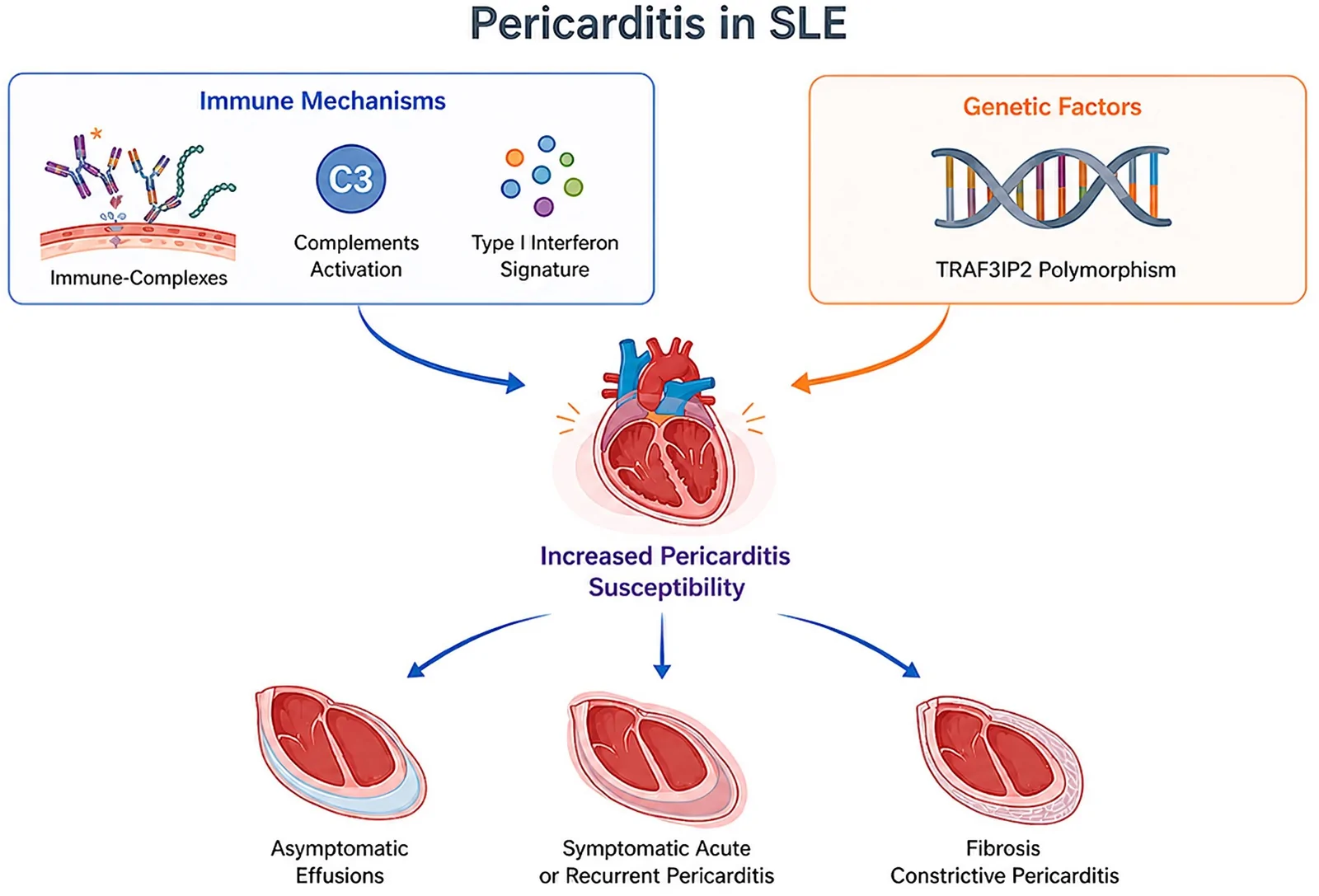
Predisposing factors and immune mechanisms leading to pericarditis in SLE.

Overall, pericardial involvement in SLE, driven by systemic immune activation, presents with a variety of clinical manifestations ranging from asymptomatic to life-threatening complications.

### Endocardium and heart valves

The first description of cardiovascular manifestations in SLE dates to 1924, when Libman and Sacks described atypical sterile thrombotic endocarditis in SLE patients [24]. All four cardiac valves can be affected, with verrucous vegetations or leaflet thickening most commonly of the mitral and aortic valve, leading to regurgitation. These lesions are sterile and typically develop secondary to chronic inflammation, and may progress to fibrosis [9, 25].

The pathogenesis of LSE is multifactorial and begins with endothelial injury. This initial insult promotes immune complex deposition and complement activation on valvular surfaces. Antiphospholipid antibodies (aPLs)—particularly anti-cardiolipin and anti-β2-glycoprotein I—further amplify this process by binding to phospholipid-binding proteins on endothelial cells and platelets. This interaction promotes a local prothrombotic environment, favouring sterile platelet–fibrin thrombus formation and vegetation growth [16, 26]. Immunofluorescence studies of explanted valves have demonstrated subendothelial immunoglobulin and complement deposition, together with aPL co-localization, supporting immune complex-mediated valvular injury [16]. More recently, neutrophil extracellular traps (NETs) have been implicated as an additional pathogenic mechanism. Active NET formation has been identified both in mitral valve tissue and in the circulation of SLE patients with LSE. NET remnants co-localize with IgG, C1q, and C3c deposits, suggesting a direct contribution to valvular injury. Importantly, NET-mediated damage may occur even in the absence of detectable aPLs, representing a potential seronegative pathogenic pathway [27].

Postmortem studies document endocardial changes—endothelial proliferation, inflammatory cell infiltration, and fibroblast proliferation—in over 50% of SLE patients, despite clinically apparent valve disease being present in fewer than 10%–20% [28]. Echocardiographic studies confirm a high subclinical burden: valve abnormalities are detected in 61% of aPL-positive SLE patients, underscoring the particularly high valve disease burden in this serological subset [29].

Valve involvement may be asymptomatic or manifest as heart failure, arrhythmias, or thromboembolic events—including stroke and peripheral organ infarction—particularly in patients with concomitant aPLs [30]. Echocardiography remains the cornerstone of diagnosis, enabling detection of valvular thickening, regurgitation, and characteristic vegetations [31].

At the genetic level, MBL2 lectin-pathway polymorphisms have been associated with cardiac valve lesions in SLE although this association appears to be partially confounded by coexisting antiphospholipid syndrome (APS) and requires independent validation [32].

aPLs are detected in ∼37% of unselected SLE patients and are associated with a markedly increased risk of cardiac involvement extending beyond LSE [33]. In patients with APS, cardiac manifestations extend beyond LSE and include coronary thrombosis and myocardial infarction, intracardiac thrombosis, thromboembolic or microvascular pulmonary hypertension, and recurrent ischaemic myocardial injury [34]. aPL positivity is also associated with increased cerebrovascular risk in SLE [34]. The 2023 ACR/EULAR APS classification criteria formally recognize this cardiac involvement by including a dedicated cardiac valve domain, supporting the view that valvular disease represents a primary manifestation of APS [35].

### Myocardium

For long, endocarditis and pericarditis dominated research and clinical interest in SLE. However, since its recognition, myocardial involvement has emerged as a serious complication of SLE. While myocarditis is certainly less common than pericarditis, with a reported prevalence of 5%–14%, it remains clinically significant, most often affecting young African–American females [36], consistent with the broader epidemiologic pattern of SLE, which disproportionately affects this population due to a combination of genetic susceptibility and socioeconomic determinants [37]. Its complex pathogenesis, resulting from dysregulated interactions between innate and adaptive immunity, along with its diverse clinical presentations and outcomes, have positioned lupus myocarditis at the forefront of ongoing research in SLE-related cardiovascular disease [13, 38].

What distinguishes lupus myocarditis from other SLE target-organ manifestations is the convergence of cardiac-specific mechanisms with broader systemic immune and thromboinflammatory pathways. A central feature is the defective thymic tolerance to α-myosin heavy chain, which permits the escape of autoreactive T cells capable of selectively targeting the myocardium [39]. Supporting the concept of primary cardiac autoimmunity, murine lupus models demonstrate myocardial T-cell infiltration preceding the onset of glomerulonephritis, indicating that myocardial inflammation is not simply secondary to cardiorenal dysfunction [40].

In parallel, cardiac-enriched autoantigens, including DIP2A and LMO7, have been identified as targets of lupus autoantibodies, directly impairing cardiomyocyte metabolism and fibroblast function independently of immune-cell infiltration [41]. Anti-Ro/SSA antibodies are independently associated with late gadolinium enhancement (LGE) on CMR, while elevated IL-18 correlates with myocardial—but not renal—involvement, suggesting that cardiac inflammatory pathways are at least partly distinct from those driving LN [42].

Lupus anticoagulant may further contribute through subclinical microvascular thrombosis, providing an additional ischaemic mechanism of myocardial injury [43]. NETs represent another potential link between thromboinflammation and fibrosis. Experimental studies demonstrate that NET-associated tissue factor and IL-17 promote fibroblast activation, while NETs have already been identified in Libman-Sacks valve lesions, supporting the hypothesis that similar mechanisms may contribute to myocardial fibrosis, although direct evidence in cardiac tissue is still lacking [27, 44, 45].

Collectively, these findings suggest that lupus myocarditis arises through a combination of cardiac-specific immune tolerance defects, autoantibody-mediated injury, thromboinflammation, and fibrosis, although additional tissue-specific mechanisms remain to be elucidated.

Lupus myocarditis often occurs early in the course of SLE and seems to be the most challenging cardiac complication to diagnose. In a series of 457 SLE patients, the condition was detected in 6% of the patients, mostly during the active disease (SLE disease activity index ≥12). Notably, 68% of SLE patients with lupus myocarditis also had LN [46, 47]. Overall mortality in these series was substantial, reaching 43% in one retrospective tertiary-referral cohort [28]; a complementary hospital series reported 25% in-hospital mortality but complete myocardial recovery in 64% of survivors [29]. Myocardial disease in SLE may present as acute myocarditis or chronic inflammatory cardiomyopathy. Clinical symptoms of acute insult range from subclinical to fulminant heart failure, including acute and severe chest pain, dyspnoea, tachycardia, haemodynamic instability, ventricular arrhythmias, and elevated high sensitivity troponin. Acute myocardial inflammation is the histologic hallmark [48–50]. In contrast, chronic inflammatory cardiomyopathy is a persistent/chronic low-grade inflammatory condition that runs silently and sub-clinically, and may present with heart failure, ventricular dysfunction, dilated and/or hypokinetic cardiomyopathy, and increased risk for arrhythmias. It can follow acute myocarditis or arise independently due to chronic immune activation [51].

Although the incidence of clinically overt myocarditis has declined with improved disease management, subclinical myocardial disease remains a significant and often undetected burden. Chronic inflammatory cardiomyopathy may still develop independently of acute events, primarily driven by immune dysregulation [52]. Therefore, there is a clear need for early detection of myocardial injury using diagnostic modalities capable of identifying subclinical disease.

Emerging evidence suggests that complement activation contributes to subclinical myocardial injury in SLE. Reduced serum C3 levels correlate inversely with CMR markers of myocardial inflammation and diffuse fibrosis, including native T1 mapping, extracellular volume fraction (ECV), T2 signal, and impaired longitudinal strain. In parallel, low serum C4 has emerged as an independent predictor of left ventricular myocardial deformation abnormalities on speckle-tracking echocardiography (STE). Together, these findings identify complement activation as a measurable link between systemic immune dysregulation and subclinical myocardial injury in SLE [53, 54].

No published genetic association study has used SLE myocarditis, inflammatory cardiomyopathy, or direct myocardial injury as a dedicated endpoint. As a result, the genetic contribution to myocardial risk in SLE remains indirect and is largely inferred from studies of ischaemic cardiovascular events, interferon pathway biology, and the broader autoimmune susceptibility landscape [55–58].

Genome-wide analyses have identified SLE-associated cardiovascular risk loci, including IL-19 and SRP54-AS1, which are linked to ischaemic events independently of traditional risk factors. These associations appear to reflect prothrombotic mechanisms—likely mediated through IL-10-driven B-cell activation and aPL accumulation—rather than primary myocardial inflammation [57, 58].

Interferon-pathway genetics provides a more mechanistically relevant framework. Gain-of-function mutations in IFIH1, encoding the innate RNA sensor MDA5, lead to constitutive type I interferon signalling and have been associated with a broad spectrum of autoimmune phenotypes that may include myocarditis. In parallel, common SLE susceptibility variants across IFIH1, IRF5, STAT4, and related loci are thought to amplify systemic inflammatory burden and increase overall cardiac risk, without conferring myocarditis-specific susceptibility [55, 56].

Overall, current evidence suggests that myocardial vulnerability in SLE reflects the cumulative effects of a sustained interferon-driven inflammatory state and a prothrombotic genetic milieu, rather than distinct cardiac-specific genetic determinants. This question remains unresolved and will require genome-wide studies incorporating dedicated myocardial phenotyping.

## DIAGNOSTIC APPROACH OF CARDIAC INVOLVEMENT IN SLE

### Clinical findings—electrocardiogram and chest X-ray

Cardiac involvement in SLE has heterogeneous clinical presentation, while routine diagnostic tools, including electrocardiogram (ECG) and chest X-ray (CXR), provide an initial evaluation but are often non-specific (Table 2). Pericarditis typically presents as intense, pleuritic chest discomfort alleviated by sitting forward, accompanied by fever, dyspnoea, and a pericardial friction rub; substantial effusions may lead to tamponade, resulting in hypotension, tachycardia, and jugular venous distension [59]. A less pronounced or non-specific appearance may occur in cases of endocardial or myocardial involvement. Particularly, it may manifest as fatigue, exertional dyspnoea, palpitations, or indicators of valve failure, including systolic murmurs resulting from mitral or aortic regurgitation, in the case of endocarditis [60]. Valvular vegetations may even serve as a source of emboli, resulting in ischaemic events, especially in individuals who also have aPLs [30]. Unfortunately, clinically evident myocarditis is even more uncommon, affecting <10% of individuals with SLE. However, it can be associated with serious consequences, such as acute heart failure, cardiogenic shock, conduction blocks, and, in severe cases, life-threatening ventricular arrhythmias [61].

**Table 2:** Clinical and routine diagnostic findings in SLE-associated cardiac disease.

Category	Findings
**Clinical**	Pleuritic chest pain relieved by sitting forward Fever Dyspnoea and reduced exercise tolerance Fatigue Palpitations Pericardial friction rub Hypotension, tachycardia, and jugular venous distension in cases of tamponade Systolic murmurs related to mitral or aortic regurgitation Signs of acute heart failure or cardiogenic shock in severe cases Thromboembolic or ischaemic events, particularly in the presence of aPLs
**ECG**	Diffuse ST-segment elevations PR-segment depression T-wave inversion Low-voltage QRS complexes Non-specific ST-T abnormalities Conduction disturbances Atrial or ventricular arrhythmias
**CXR**	Cardiomegaly Pulmonary congestion Frequently normal with limited sensitivity for detecting cardiac involvement

ECG and CXR findings, much like clinical presentation, are variable and usually non-specific. Disorders such as diffuse ST-segment elevations, PR-segment depression, T-wave inversion, and low-voltage QRS complexes are typical in pericarditis [62]. ST-T and conduction abnormalities or arrhythmias are even less specific changes, evident in myocarditis or endocarditis [63]. CXR has limited diagnostic value; however, cardiomegaly or pulmonary congestion are indicative of moderate-to-large pericardial effusion or heart failure respectively [64]. Overall, clinical and routine diagnostic findings are subtle, and further imaging modalities are required for definitive diagnosis.

### Echocardiography

During the last years, research focus has gradually shifted from autopsy studies and endomyocardial biopsies (EMBs) towards non-invasive imaging methods in living patients with SLE in order to diagnose lupus carditis and especially myocarditis early and to assess prognosis [49, 65]. Conventional transthoracic echocardiography (TTE) is a useful examination to demonstrate established pericardial or valvular involvement but not early or subtle myocarditis. In a retrospective study of 76 SLE patients diagnosed between 2008 and 2017, echocardiographic findings revealed cardiac involvement in 52%, most often pericarditis and valvular abnormalities. Myocardial involvement was detected in only 12.5%, with no signs of acute myocarditis [66].

Echocardiographic modalities, including scar imaging echocardiography with ultrasound multi-pulse scheme (eSCAR) and STE with global longitudinal strain (GLS) assessment, are more sensitive for early or subclinical myocardial involvement. STE is a sophisticated method that can identify and label speckle patterns within the myocardium using an algorithm. It reproduces either segmental strain as regional deformation or global longitudinal strain as global deformation [67]. STE can assess segmental and global myocardial deformation and has high specificity for myocarditis in non-lupus patients.

In SLE, STE can detect impaired GLS even in patients with preserved ejection fraction, highlighting its value in early detection [68, 69]. Recent studies using eSCAR and STE found myocardial scars in 19% of SLE patients, localized mainly to the inferoseptal segment, and correlated with disease activity. All eSCAR-positive patients had impaired GLS, but conventional echocardiography often missed these findings, underscoring the need for advanced techniques. Positivity was associated with higher anti-dsDNA antibodies and increased flare risk, suggesting its utility for cardiac surveillance and relapse prediction [70].

Despite these advances, echocardiographic techniques cannot accurately detect myocardial injury or distinguish between underlying pathological processes (oedema, fibrosis, etc.).

### Cardiac magnetic resonance

In this context, CMR has emerged as the most comprehensive, non-invasive imaging modality for the evaluation of myocardial involvement in SLE. CMR enables quantification of biventricular volumes, myocardial mass, and function, while providing tissue characterisation capabilities for the detection of myocardial injury, inflammation, and scarring [71]. The widespread use of artificial intelligence and advanced algorithms has further enhanced the diagnostic capabilities of CMR in systemic diseases [11, 72, 73]. CMR is now the non-invasive reference standard for myocarditis diagnosis, as per the 2020 Society for Cardiovascular Magnetic Resonance guidelines [74–76].

Studies since the late 1980s have shown that SLE patients have higher native T1 relaxation times than healthy controls or patients with other cardiomyopathies, especially during active disease [77]. T2 relaxation time also serves as a marker of activity, normalizing with remission [78]. In addition, mapping techniques and early gadolinium enhancement correlate with disease activity as measured by the European Consensus Lupus Activity Measurement (ECLAM) index [79]. Advancements in mapping techniques now allow precise tissue characterisation, even without the use of contrast agents [80, 81].

Prognostically, the presence and extent of LGE have emerged as independent predictors of major adverse cardiovascular events (MACE) in SLE. LGE positivity (HR 3.75, *P* = .007) and greater LGE burden (HR 3.74, *P* = .019) have both been independently associated with adverse outcomes, while the combination of LGE findings with organ damage indices identifies a particularly high-risk subgroup [82]. In parallel, mapping-derived parameters, including native T1 and ECV, also correlate with disease activity indices and complement biomarkers across SLE-specific CMR series [83] (Table 3). Despite these advances, data specifically evaluating CMR patterns and their prognostic significance in patients with active LN—distinct from SLE without renal involvement—remain limited.

**Table 3:** Key SLE-specific CMR studies evaluating myocardial involvement.

Study	*N* (LN/SLE)	Main CMR abnormalities	Correlations reported	Outcomes reported
Abdel-Aty *et al*., 2008	–/20	T2 elevation and EGE	Correlated with ECLAM disease activity index	None reported
Zhang *et al*., 2015	–/24	Higher T2 times in T2-mapping	Subclinical inflammation detected in asymptomatic SLE patients	None reported
Puntmann *et al*., 2013	15/33	Elevated native T1; subclinical perimyocardial involvement	Native T1 was independent predictor of SLE diagnosis; no correlation with eGFR or proteinuria	None reported
Seneviratne *et al*., 2016	–/41	LGE in 37% (15/41); interventricular septum-predominant; intramural/inflammatory distribution	No correlation with disease activity or SLE duration	Extensive LGE (>15% LV mass) associated with diastolic dysfunction and reduced exercise capacity
Burkard *et al*., 2018	30 SLE (30% with renal disorder)	13/30 abnormal CMR; all detected LGE (9/13) non-ischaemic; stress perfusion deficits in 13%	LGE associated with microalbuminuria; stress perfusion deficits associated with renal ACR criterion	None reported
Feng *et al*., 2023	37/74	LGE in 52.7% (39/74): septum-only 27%, LVFW-only 12%, both locations 14%; LGE degree comparable to non-autoimmune cardiomyopathy controls, global myocardial function impaired in all SLE vs healthy controls	LGE associated with antiphospholipid syndrome, elevated NT-proBNP and cardiac troponin I; LVFW LGE predicts cardiac mortality (HR 6.35, *P *= .007); LGE combined with organ damage score (SDI) identifies highest-risk subgroup	LGE independently predicts MACE
Huang *et al*., 2025	15/42	Elevated native T1, ECV, and T2; GCS identifies subclinical myocardial involvement	C3 inversely correlated with native T1, ECV, and T2; ESR independently correlated with ECV	None reported

Abbreviations: ACR, American College of Rheumatology; CMR, cardiac magnetic resonance; ECLAM, European Consensus Lupus Activity Measurement; ECV, extracellular volume fraction; EGE, early gadolinium enhancement; ESR, erythrocyte sedimentation rate; GCS, global circumferential strain; LGE, late gadolinium enhancement; LV, left ventricle; LVFW, left ventricular free wall; MACE, major adverse cardiovascular events; *N*, sample size; NT-proBNP, N-terminal proB-type natriuretic peptide; SLE, systemic lupus erythematosus; –, not reported.

In an attempt to better describe and classify CMR findings in myocarditis, the International Consensus Group on CMR Diagnosis of Myocarditis introduced the Lake Louise criteria (LLC), based on a constellation of clinical symptoms and CMR findings. The LLC, initially introduced in 2009 and revised in 2018, provide a standardised framework for diagnosis by integrating functional disorders and tissue pathology [84, 85]. It should be noted, however, that the LLC were developed and validated in general myocarditis populations—predominantly viral and immune-mediated—and have not been prospectively assessed in dedicated SLE cohorts; therefore, their application in SLE relies largely on extrapolation rather than direct evidence. At the same time, novel techniques aimed at enhancing diagnostic accuracy, such as LGE, T1/T2 mapping, and strain imaging, have been widely adopted in clinical practice.

According to the LLC 2009, the diagnosis of myocarditis could be established in suspected cases when at least two of the following criteria were present: (i) regional or global myocardial signal intensity (MSI) increase in T2-weighted images compared to the skeletal muscle, suggestive of myocardial inflammation and oedema, (ii) increased global myocardial early gadolinium enhancement (EGE) ratio between myocardium and skeletal muscle in gadolinium-enhanced T1-weighted images, and (iii) presence of at least one focal lesion with non-ischaemic regional distribution in inversion recovery (IR)-prepared gadolinium-enhanced T1-weighted images, defined as LGE [51]. Although the above criteria proved useful in clinical practice, their limited accuracy in cases with mild myocardial inflammation and the continuing development of more sophisticated methods, such as T1 and T2 mapping techniques, led to the revision of LLC in 2018. The revised criteria incorporate T1-based and T2-based markers, with the presence of at least one criterion from each category supporting the diagnosis of acute myocardial inflammation [86]. Compared to the 2009 LLC, implementation of mapping techniques increased the diagnostic accuracy of CMR in detecting myocarditis, reaching up to 95% for T1 mapping and 88% for T2 mapping [87]. Most importantly, CMR may detect subclinical myocardial abnormalities in 30%–55% of cases [88, 89]. Overall, the integration of advanced CMR techniques has markedly enhanced the ability to identify even mild myocardial injury, supporting its pivotal role in early recognition and risk stratification in SLE.

### Proposed clinical approach in SLE and LN

Given the limited and heterogeneous evidence available, a pragmatic approach to the assessment of cardiac involvement in patients with SLE and LN may be considered. In clinical practice, baseline TTE may be appropriate in patients with established SLE, particularly in the presence of LN, high disease activity, or cardiovascular risk factors.

Advanced imaging modalities, including STE or CMR, may be considered in patients with suggestive clinical features, elevated cardiac biomarkers (e.g. troponin or natriuretic peptides), persistent disease activity, or abnormal findings on initial echocardiographic evaluation. In particular, CMR may be valuable for detecting subclinical myocardial involvement when conventional imaging is inconclusive.

In patients with LN, closer cardiovascular surveillance may be warranted, especially during periods of increased disease activity, following major therapeutic changes, or in the presence of progressive structural or functional cardiac abnormalities. Although evidence-based recommendations are lacking, repeat imaging may be considered on a case-by-case basis, guided by clinical evolution and multidisciplinary evaluation. A conceptual approach to the assessment of cardiac involvement in patients with SLE and LN is presented in Fig. 3.

**Figure 3:**
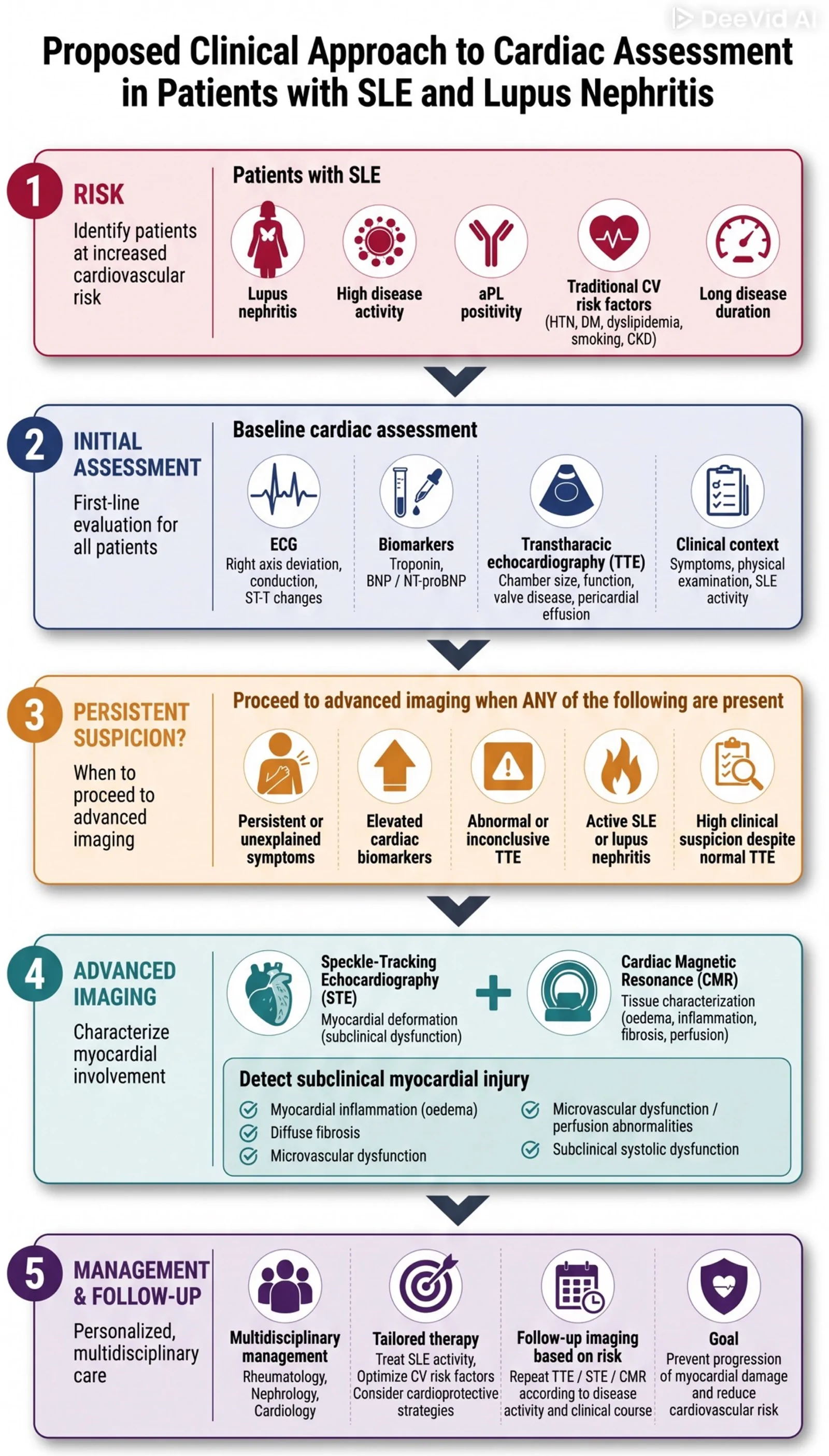
Proposed clinical approach to the assessment of cardiac involvement in patients with SLE and LN.

Further studies are needed to establish evidence-based screening and risk-stratification strategies and to identify clinical, immunological, or biomarker profiles that may justify early use of advanced imaging. Until such data become available, the proposed approach should be regarded as a conceptual framework requiring individualized clinical assessment.

### Histopathology findings

EMB is an invasive diagnostic method associated with increased risk of severe complications and is certainly not performed on a regular basis to estimate cardiovascular complications of systemic diseases [90]. However, prior to the development of high-quality imaging methods, EMB represented the only definitive method to diagnose myocardial involvement in SLE.

Early histopathological evidence of myocardial involvement in SLE was derived primarily from postmortem studies. These demonstrated necrotic tissue injury, characterized by fibrinoid necrosis and irreversible nuclear damage like pyknosis and karyolysis. Myocardial fibres were not regularly affected, often exhibiting a patchy degeneration with disruption of nucleonic membranes, resembling inclusion bodies of lupus erythematosus (LE) cells [28]. Myocardial fibrosis was present but not frequent.

These observations subsequently prompted the evaluation of EMB in living patients with SLE. Subsequent studies using EMB in living patients, taken from the right ventricle of SLE patients, usually displayed myocarditis, drug toxicity, or non-specific findings; importantly, a series of myocardial biopsies proved that early histological alterations consist of inflammatory interstitial infiltration, while nuclei degeneration of the monocytes or lymphocytes could produce Feulgen-positive haematoxylin bodies leading to fibrinoid accumulation. Ischaemic injury, necrosis, myocyte damage, chronic active myocarditis, and also perivascular inflammation, endothelial damage, thrombi, or even haemorrhagic myocarditis are indications of SLE-induced small vessel disease. At the very early stages, fine granular IgG and C1q deposition may be the only finding seen in immunofluorescence staining [60, 61] (Fig. 4).

**Figure 4:**
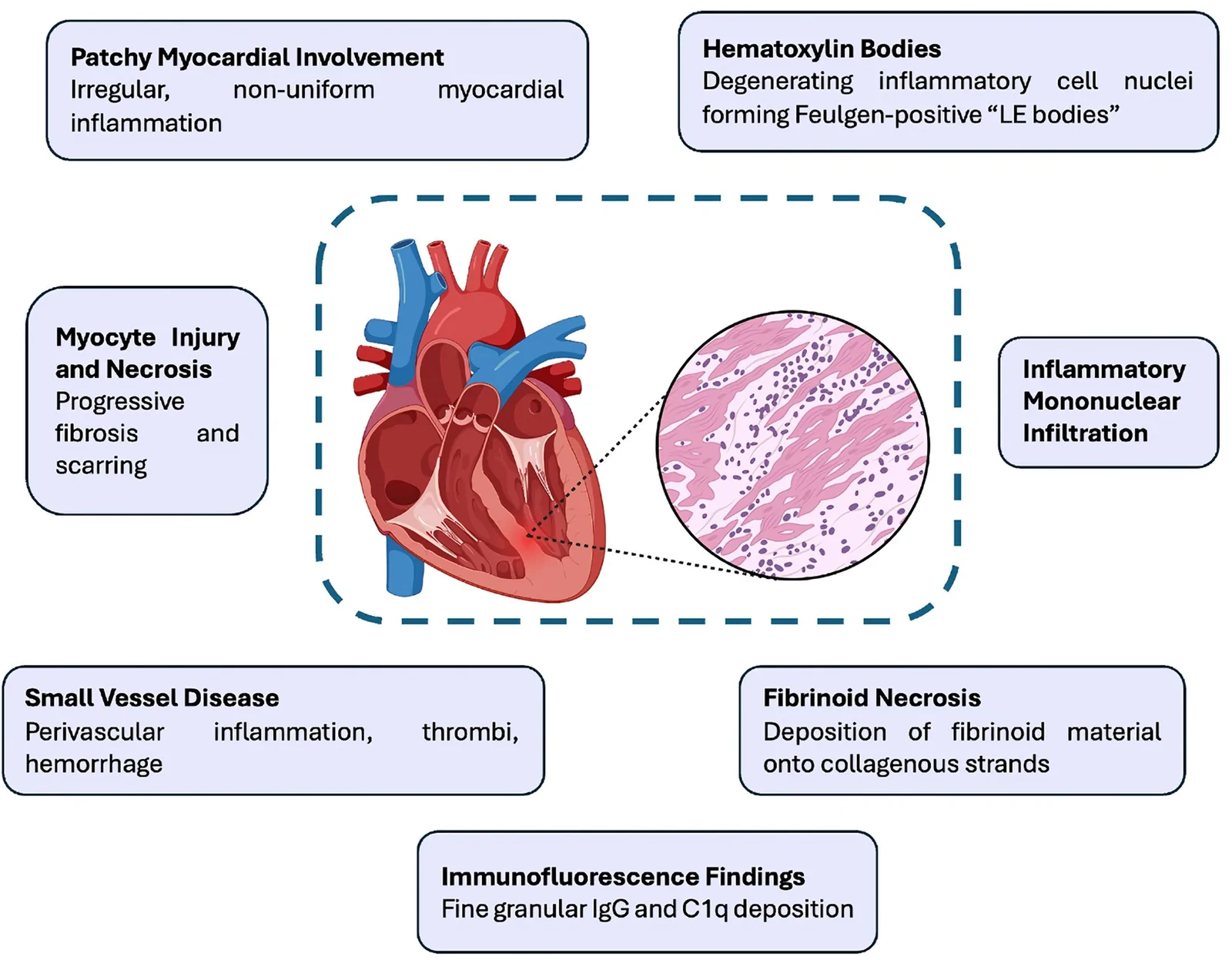
Lupus myocarditis in SLE: histopathological findings. Myocardial involvement in lupus myocarditis is characteristically patchy and subject to sampling error; a negative EMB therefore does not exclude active disease. Created in BioRender. MOYSIDOU, E. (2026) https://BioRender.com/fcmmtww

Despite its diagnostic value, EMB has certain limitations. The patchy distribution of myocardial involvement in SLE reduces its sensitivity, while histological findings are often non-specific and may overlap with other causes of myocarditis [91, 92]. As a result, EMB lacks both sensitivity and specificity for routine diagnostic use in this setting.

Overall, EMB has traditionally been considered the histological gold standard for confirming myocarditis. However, its invasive nature and associated risks—including perforation, arrhythmias, conduction disturbances, and other procedural complications—limit its routine use. It is generally reserved for selected clinical scenarios where non-invasive methods cannot provide a clear diagnosis or when histology would significantly alter therapeutic management [50, 93, 94]. In clinical practice, EMB findings in lupus myocarditis are often non-specific and largely indistinguishable from viral myocarditis on light microscopy [49]. As a result, immunosuppressive therapy is most commonly escalated empirically based on CMR findings—particularly the presence of LLC and abnormal T1/T2 mapping—rather than histological confirmation. EMB is therefore typically reserved for selected cases, primarily to exclude viral persistence prior to intensification of immunosuppression (e.g. IVIG or cyclophosphamide) or to support reclassification as virus-negative, immune-mediated myocarditis when patients fail to respond to conventional heart failure therapy [93].

### Lupus nephritis as a determinant of cardiovascular and myocardial involvement

LN appears to represent not only a marker of systemic disease severity but also a potential contributor to myocardial injury through both renal and immune-mediated pathways. Evidence from meta-analysis consistently demonstrates that patients with LN have an almost two-fold higher risk of cardiovascular events than SLE patients without nephritis (RR 1.75; 95% CI 1.13–2.70), and more than double that of the general population (RR 2.35; 95% CI 1.95–2.84) [95]. This is consequently translated into differences in cardiovascular mortality rates, reaching 11.7 per 1000 patient-years in LN versus 3.6 per 1000 patient-years in non-nephritic SLE [96], highlighting the clinical urgency of early cardiovascular assessment in patients with renal involvement [97].

The mechanisms underlying this heightened cardiovascular burden can be divided into two complementary but distinct pathways. The first is shared with any cause of chronic kidney disease (CKD) namely sodium and fluid retention, activation of the renin-angiotensin-aldosterone system (RAAS), and systemic hypertension that together promote concentric left ventricular hypertrophy (LVH) and diastolic dysfunction, the characteristic haemodynamic phenotype of CKD-associated cardiomyopathy [98]. Additional contributors include CKD-related anaemia, leading to increased cardiac output demands and eccentric ventricular remodelling [99], and secondary hyperparathyroidism, which accelerates vascular calcification, with direct myocardial injury [100]. The second pathway is specific to the immunological milieu of SLE and LN: circulating immune complexes, complement activation, type I interferon signalling, and antibody-mediated endothelial injury drive myocardial inflammation and non-ischaemic fibrosis independently of haemodynamic conditions (pressure or volume). Evidence from CMR studies supports this concept. Among SLE patients without known coronary artery disease (CAD), 43% demonstrated abnormal CMR findings, with all cases of LGE exhibiting non-ischaemic pattern consistent with inflammatory rather than infarct-related myocardial injury [101]. Furthermore, LGE was significantly more prevalent in patients with microalbuminuria (33.3% vs. 9.5%, *P* = .048), while stress perfusion deficits, indicative of microvascular ischaemia, were strongly associated with renal involvement as an ACR criterion (80% vs. 22%, *P* = .011) [101]. Although these findings do not establish causality, they suggest that renal involvement, even before the development of overt LN, may be associated with myocardial injury through mechanisms beyond haemodynamic overload alone.

Multiparametric CMR mapping has further delineated the inflammatory-fibrotic cardiac phenotype. SLE patients with subclinical myocardial involvement, compared to asymptomatic SLE patients with normal CMR findings, demonstrate elevated native T1 values (reflecting diffuse inflammation or fibrosis), increased ECV, and elevated T2 (reflecting myocardial oedema). Notably, complement component C3—a major mediator of immune-mediated organ damage in LN—negatively correlated with native T1 (*r* = −0.414, *P* = .006), ECV (*r* = −0.404, *P* = .008), and T2 (*r* = −0.397, *P* = .009), as well as with global circumferential and longitudinal strain, supporting an association between complement activation and diffuse fibrotic myocardial injury. Furthermore, erythrocyte sedimentation rate independently correlated with ECV (*r* = 0.309, *P* = .046), reinforcing the concept of immune-mediated myocardial injury pattern [53]. Beyond multiparametric CMR, STE has also provided evidence supporting an immune-mediated pattern of myocardial injury. A 2025 Swedish cross-sectional study of 55 patients with SLE, including a dedicated LN subgroup, demonstrated that complement protein C4 was the sole independent predictor of left ventricular mechanical dispersion (LVMD) on STE (standardised *β* = −0.45, *P* < .001), whereas neither estimated glomerular filtration rate (eGFR) nor urine albumin-to-creatinine ratio independently predicted LVMD [54]. These findings suggest that myocardial contractile dyssynchrony is more closely associated with complement-mediated inflammation than with renal haemodynamic impairment, thereby providing echocardiographic evidence that complements the CMR findings linking complement dysregulation with diffuse myocardial injury.

The histological class of LN represents another potentially important determinant of cardiovascular risk. In a large real-world cohort of 3732 SLE patients, LN was independently associated with CAD after multivariate adjustment (OR 1.47; 95% CI 1.07–2.02; *P* = .017), while further analysis demonstrated important heterogeneity across different nephritis classes: mixed class III/V nephritis (OR 2.23; 95% CI 1.09–4.62; *P* = .028) carried the highest CAD odds followed by class III (focal proliferative; OR 1.98; 95% CI 0.95–4.12), while class IV (diffuse proliferative) and class V (membranous) did not reach statistical significance [102]. However, the absence of important clinical variables, such as proteinuria, eGFR, and complement levels, precluded a more comprehensive interpretation of the findings.

At the subclinical level, STE provides further evidence of a distinct myocardial phenotype in LN. Compared with patients with extra-renal SLE, those with LN exhibited significantly impaired GLS (GLS: −17.61% vs. −19.36%, *P* < .001) and increased LVMD (LVMD: 42.96 ms vs. 35.62 ms, *P* < .001), indicating more pronounced subclinical myocardial dysfunction [103]. Moreover, 24-h urinary proteinuria independently predicted LVMD on multivariable analysis (*β* = 0.793, *P* < .05) [103], suggesting an independent association between proteinuria severity and myocardial mechanical dyssynchrony. The reproducibility of these observations is underscored by an independent Swedish cross-sectional study, which similarly identified increased LVMD in patients with LN despite using a different comparator population (healthy controls rather than extra-renal SLE) [54]. While GLS remained preserved in that cohort, the concordant elevation in LVMD across two independent studies suggests that myocardial mechanical dyssynchrony may represent one of the earliest and most consistent manifestations of subclinical cardiac involvement in LN. Furthermore, the Swedish investigators demonstrated concomitant right ventricular impairment, reflected by reduced tricuspid annular plane systolic excursion (TAPSE) and right ventricular free wall strain [54], suggesting that the subclinical cardiac phenotype of LN may extend beyond the left ventricle to involve biventricular myocardial dysfunction.

In parallel with these imaging abnormalities, renal disease further amplifies cardiovascular risk through metabolic and haemodynamic mechanisms. Nephrotic-range proteinuria promotes a characteristic nephrotic dyslipidaemia caused by urinary loss of proteins involved in lipoprotein metabolism, resulting in markedly higher odds of dyslipidaemia in LN than in non-nephritic SLE [96]. The consequent elevation in LDL cholesterol and triglycerides accelerates endothelial dysfunction and atherosclerotic plaque formation, particularly in younger patients with otherwise low baseline cardiovascular risk. Hypertension, affecting up to 80% of LN patients [96], further compounds this risk through persistent RAAS activation, sodium retention, and progressive renal parenchymal damage, thereby promoting pressure-overload LVH.

Collectively, these findings suggest that LN may be associated with a characteristic subclinical myocardial phenotype, the imaging features of which closely mirror the CMR abnormalities discussed earlier in this review. Patients with LN who develop myocarditis or subclinical myocardial involvement predominantly demonstrate elevated T2 values together with diffuse native T1 and ECV abnormalities, reflecting active myocardial oedema superimposed on progressive interstitial fibrosis [53, 80]. The ischaemic component—manifesting as stress perfusion deficits rather than epicardial infarct-pattern LGE—appears to be driven by microvascular dysfunction, a product of endothelial injury, immune complex deposition, and the haemodynamic consequences of long-standing hypertension and nephrotic dyslipidaemia [101].

These distinct imaging patterns may also have therapeutic implications. While non-ischaemic myocardial abnormalities primarily reflect immune-mediated injury and may prompt consideration of underlying immune-mediated activity and optimization of immunosuppressive therapy, stress perfusion abnormalities highlight the importance of comprehensive cardiovascular risk reduction, including RAAS blockade, lipid-lowering therapy, and optimal blood pressure control where appropriate [96]. Treatment-related factors should likewise be considered, as prolonged corticosteroid exposure and calcineurin inhibitors may further amplify cardiovascular risk [104, 105]. Consequently, steroid-sparing strategies incorporating mycophenolate mofetil, belimumab, voclosporin, or obinutuzumab may indirectly reduce cardiovascular burden by improving renal disease control and limiting cumulative corticosteroid exposure [106].

Despite these advances, prospective imaging studies directly comparing biopsy-confirmed LN with non-LN SLE while accounting for kidney function, proteinuria, histological class, and cumulative immunosuppressive exposure remain lacking. Whether LN independently increases the prevalence of non-ischaemic myocardial injury and whether CMR mapping abnormalities correlate with nephritis class or disease activity are key unanswered questions. Addressing these gaps will require prospective multicentre studies integrating longitudinal renal biomarkers with comprehensive cardiac phenotyping using CMR and STE.

## CONCLUSIONS

Cardiac involvement in SLE represents a serious clinical manifestation, encompassing a broad spectrum of abnormalities affecting any cardiac structure. Among these, myocardial involvement remains particularly challenging due to its frequently silent presentation and the limited sensitivity of conventional diagnostic approaches. Growing evidence supports the central role of immune-mediated mechanisms in its pathogenesis, leading to inflammation, fibrosis, and adverse cardiac remodelling; however, a substantial translational gap persists. Current clinical approaches rely largely on the detection of established structural or functional abnormalities, thereby limiting opportunities for early intervention. CMR has emerged as a pivotal tool for non-invasive assessment of myocardial injury, enabling the detection of subclinical disease. The integration of advanced imaging techniques into clinical evaluation is expected to facilitate earlier diagnosis and improve patient management. These considerations highlight the need for a shift from descriptive, lesion-based phenotyping towards a mechanism-driven approach to cardiac risk stratification in SLE.

Emerging technologies may provide a framework to support this transition. The integration of high-throughput multi-omics platforms with artificial intelligence and machine learning may redefine lupus-associated cardiac disease by resolving molecular heterogeneity and identifying discrete immunopathological endotypes [107, 108]. Data-driven classifications of SLE have identified distinct molecular profiles, including interferon-driven, neutrophil-dominated, plasmablast-mediated, and complement-associated signatures, each linked to specific clinical phenotypes, such as active LN and systemic inflammation.

These molecular patterns may extend beyond renal involvement and contribute to distinct cardiovascular manifestations. These observations raise the hypothesis that interferon- and neutrophil-driven signatures may predispose to diffuse inflammatory myocardial involvement, whereas complement- and vascular-related pathways may be more closely linked to accelerated atherosclerosis and thrombotic complications. Consistent with this concept, transcriptomic studies in LN have demonstrated conserved, cross-tissue molecular signatures reflecting systemic immune activation, which may promote endothelial dysfunction and myocardial injury even in the absence of overt cardiac symptoms.

Integrating such molecular profiles with advanced cardiac imaging—particularly CMR-derived markers of myocardial inflammation and fibrosis—may enable more precise identification of subclinical cardiac involvement and facilitate improved risk stratification. Future studies should incorporate composite endpoints capturing both cardiac and renal outcomes, including CMR-defined myocardial abnormalities, major adverse cardiovascular events, and kidney disease progression. The implementation of these approaches in clinical practice will require coordinated efforts, including standardised imaging protocols, centralized CMR core laboratories, and integration with existing LN cohorts and biobanks.

Ultimately, bridging molecular endotyping with imaging-defined cardiac phenotypes may allow earlier detection of subclinical myocardial involvement and support the development of mechanism-driven, personalized strategies for cardiovascular risk assessment and management in patients with SLE.

## Data Availability

No new data were generated or analysed in support of this review.
